# Bio-inspired one-pot synthesis of luminescent silver nanoparticles and its significant utility as a fluorescence nano sensor for analysis of two adjunctive COVID-19 drugs

**DOI:** 10.1186/s13065-024-01335-8

**Published:** 2024-11-14

**Authors:** Yasmeen E. Mostafa, Fawzi Elsebaei, Mohammed El-Sayed Metwally

**Affiliations:** Department of Pharmaceutical Analytical Chemistry, Faculty of Pharmacy, Mansoura University, P.O. Box 35516, Mansoura, Egypt

**Keywords:** Silver nanoparticles, Green synthesis, Sensor, Quenching, Orange peel, Direct oral anticoagulants, COVID-19

## Abstract

This study reveals one-step green synthesis of plant inspired silver nanoparticles (Ag-NPs). The synthesis procedure relies on the bio-reduction of Ag^+^ to Ag^0^ using orange waste (orange peel) extract as cheap, readily available, sustainable, biocompatible feedstocks as a reducing and stabilizing agent. The prepared Ag-NPs passed through a full characterization procedure for better confirmation and elucidation of optical and structural properties. The fluorescence of the prepared Ag-NPs has a quantum yield of 17.15% enabling its potential use in chemical sensing of drugs. Ag-NPs are conceived to be used as a fluorescent nano sensor for sensitive, ecofriendly, rapid spectrofluorimetric determination of two recent direct oral anticoagulants, namely, rivaroxaban (RIV) and edoxaban tosylate monohydrate (EDT); COVID-19 adjunctive drugs in their raw materials and pharmaceutical tablets. The fluorescence of the prepared Ag-NPs at 333 nm ${(\uplambda }_{\text{ex}}=258 \text{nm})$ was found to be substantially quenched in existence of increasing concentrations of each drug. The quenching mechanisms were studied and explained. The validation of the method revealed linear correlation over the ranges of 0.5–10 µg/ml with an excellent regression correlation (r = 0.9999) for both drugs with minimum detection limits of 0.14 and 0.16 µg/ml for rivaroxaban and edoxaban tosylate monohydrate, correspondingly. Three different metrics were employed for verifying the greenness profile of the presented study. The findings of the greenness assessment were congruent and compatible with the green synthesis procedure, ecofriendly analysis, and the exclusion of using organic solvents and noxious materials opening an avenue for green synthesis of nanoparticles instead of chemical and physical methods.

## Introduction

The recent FDA approved direct oral anticoagulants (DOACs) have gained much attention in many clinical scenarios. The light was shed on the significance of oral anticoagulants in the prevention and management of thrombosis in various cardiovascular events. DOACs are deemed to substitute the antecedent standard anticoagulant agents; vitamin K antagonists such as warfarin and low molecular weight heparins in minimizing the hazard of thromboembolic repercussions with lower bleeding risk [1]. DOACs provide superior advantages over vitamin K antagonists as they show rapid anticoagulation activity after oral ingestion, the omission of routine INR monitoring, wide therapeutic index, less recurrent follow up and the low possibility of exhibiting drug and food interactions [2, 3]. Those privileges have provided both patients and healthcare professionals with more safe, convenient, effective alternatives to heparin [4].

Moreover, the pandemic coronavirus COVID-19 (severe acute respiratory syndrome coronavirus) has become a recent issue of concern all over the world. With the prevalence of increased mortality rate because of thromboembolic consequences secondary to COVID-19, the importance of DOACs is magnified. Thus, it is worthy to highlight on the investigation of two novel important DOACs: rivaroxaban (RIV) and edoxaban tosylate monohydrate (EDT). RIV and EDT are oral direct and selective inhibitors of factor Xa leading to anticoagulation effect. Their clinical utilization in diminishing the hazard of stroke and embolic events in non-valvular atrial fibrillation played a significant role in the reduction of mortality worldwide [1].

**Fig. 1:**
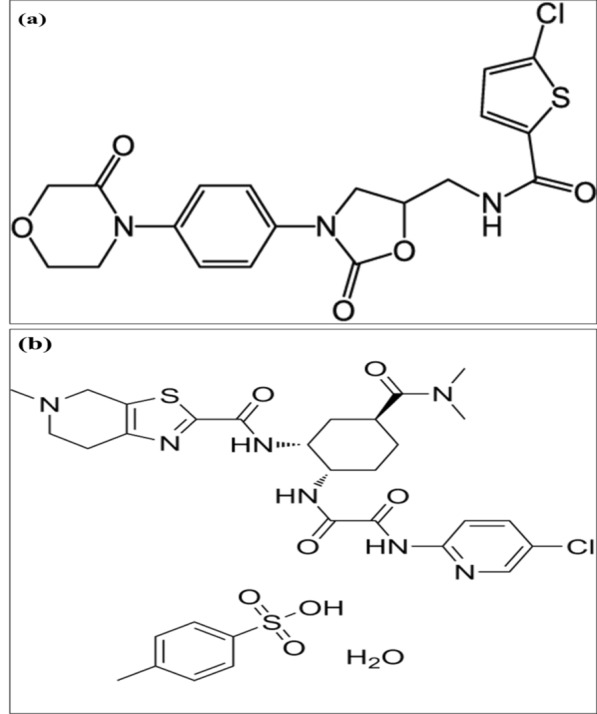
The structural formula of **a** Rivaroxaban. **b** Edoxaban tosylate monohydrate

RIV and EDT were regarded as safe and effective curative agents in management of pulmonary embolism in COVID-19. RIV was approved by the FDA in 2011 and has a half-life of about 13 h [4, 5]. It has a unique indication when co-administered with aspirin leading to decreasing critical cardiovascular thrombotic conditions such as stroke, coronary artery disease, myocardial infarction and peripheral artery disease [6]. It is unnecessary to have any cofactors for its anticoagulant action [5]. It is reported that it neither induced or inhibited cytochrome P450 (CYP) enzymes [7]. However, concurrent use of RIV and intense CYP3A4 and P-gp inducers should be cautiously administered [8]. The IUPAC name of RIV is 5-chloro-*N*-[[(5*S*)-2-oxo-3-[4-(3-oxomorpholin-4-yl)phenyl]-1,3-oxazolidin-5-yl]methyl]thiophene-2-carboxamide (Fig. 1a) [9].

EDT was approved by the FDA in 2015 in the prophylaxis from stroke in patients with atrial fibrillation and the prevention and management of consequences of venous thromboembolism. It has a half-life of 10–14 h and reaches peak plasma concentration rapidly (about 1.5 h) following oral ingestion [10]. The once daily dosing of EDT is deemed to be fundamental adjunct to the antithrombotic management drugs [11]. It has quite a low affinity for plasma proteins which is a significant aspect for hemodialytic patients [12]. Its bioavailability is not affected by food intake so, it may be given with or without taking food into account [13]. EDT showed relatively low hepatic metabolism (<4%) by cytochrome P450 (CYP450) enzymes making its potential interaction with CYP450 substrates ignorable [11]. However, potential pharmacokinetic interaction between EDT and P-glycoprotein inducers or inhibitors must be regarded to avoid their probable impact on the exposure of EDT [14]. The IUPAC name of EDT is *N*′-(5-chloropyridin-2-yl)-*N*-[(1*S*,2*R*,4*S*)-4-(dimethylcarbamoyl)-2-[(5-methyl-6,7-dihydro-4*H*-[1,3]thiazolo[5,4-c]pyridine-2-carbonyl)amino]cyclohexyl]oxamide;4-methylbenzenesulfonic acid;hydrate (Fig. 1b) [9].

The literature survey reported various techniques for the determination of RIV and EDT in raw materials and pharmaceutical dosage forms. The published techniques for RIV determination were UV spectrophotometry [15, 16], spectroflourimetry [17], HPLC [18–20], HPTLC [21], square wave voltammetry [22], UPLC [23] and LC–MS/MS [24]. Whilst for EDT, the reported techniques contained UV spectrophotometry [25, 26], spectroflourimetry [27], HPLC [28–31], UPLC–MS/MS [32], LC–MS/MS [33, 34] and capillary zone electrophoresis [35]. Despite the high sensitivity of some reported methods, they either use expensive instrumentation or large quantities of hazardous solvents opposing the principles of green chemistry [28–30] or require specialized trained personnel [32–34], which might not be attainable and compromise a limited applicability in quality control laboratories with depleted resources. Herein, the development of a rapid, simple, cost effective, sensitive, and environmentally benign quantification method of both drugs is a significant challenge.

Recently, nanotechnology and nanomaterials have emerged as a leading topic in many scientific disciplines. The rapid growing of nanomaterials has led to their contribution to the enrichment of numerous applications owing to their unique properties. The wide spread applications of nanomaterials can be established in many areas such as drug delivery [36], catalysis [37], imaging [38, 39], electrochemistry [40, 41], sensors [42, 43], optics [44], food packing [45], biomedicine [46], pharmaceuticals [47–50], molecular imaging for diagnostic purposes [51, 52] and nanomedicine [53, 54]. Nanoscience is specialized in the study of particles in the size range of 1–100 nm. Silver nanoparticles are attaining remarkable concern among other metal nanoparticles owing to their outstanding properties such as surface-enhanced Raman scattering, raised electrical and thermal conductivity, optical characteristics, chemical stability, catalytic activity, and antimicrobial activity.

Accordingly, the light is much shed on novel methods for synthesis of silver nanoparticles due to their widespread applications and crucial importance. The synthesis of Ag-NPs is accomplished using either one of two approaches: ‘top-down approach’ and ‘bottom-down approach’. The top-down approach involves the formation of small particles from bulk material by means of particle size reduction by physical methods. The bottom-down approach is based on reduction of silver nitrate into nanoparticles by organic and inorganic reducing agents through chemical and biological techniques. Capping agents are used for their stabilization [55, 56].

Enormous research is directed toward optimizing the synthesis of Ag-NPs as a priority avoiding a lot of drawbacks of the synthesis techniques. Chemical and physical techniques use relatively expensive and toxic hazardous chemicals that may produce end products detrimental to the environment and laboratory personnel [57]. The disadvantages of these traditional techniques provoke the superiority of green biosynthesis synthesis approach as a convenient alternative. They consume bioorganic materials such as microorganisms and plant biomass or plant extracts because they are ecofriendly, cost-effective, biodegradable, effective and sufficiently stable. Consequently, among biological routes, plant extract mediated synthesis of Ag-NPs outperforms microorganisms mediated synthesis because of the little biological risks and possibility of retaining cell cultures [55, 58].

For all of that, our study announces a relevant outlook for the biosynthesis of Ag-NPs from sustainable, renewable, cheap, and environmentally benign materials, orange peel extract, that in turn reduces the dependance on depleting resources and toxic chemicals. It contains vitamin C, flavonoids, acids, and volatile oils which act as bio reducing and capping agents. The biosynthesized Ag-NPs attained intense fluorescence, high water solubility, and good stability that rendered their potential utilization as a fluorescent nano sensor in pharmaceutical and biological assay. Besides, fluorimetry is a more convenient and prevailing technique for the assay of many drugs owing to its high sensitivity, selectivity, and availability in many quality control laboratories. Our suggested study overpowers the drawbacks of other reported ones by being the first fluorometric method for EDT without derivatizing agents [27]. Additionally, it is deemed as the first green fluorometric method of RIV as the reported fluorometric method used acetoxy mercuric fluorescein reagent which is very toxic by inhalation, in contact with skin and if swallowed [17].The significance of our study does not only rely on the synthesis of Ag-NPs from renewable and cheap feedstocks but also incorporating green chemistry and safe laboratory environment by the development of a fluorescent nanoprobe for the analysis of RIV and EDT. Fluorescent Ag-NPs were successfully applied for quantitative determination of two novel oral anticoagulants RIV and EDT based on the quenching behavior of the investigated drugs on the intense fluorescence of Ag-NPs with high sensitivity and accuracy. The suggested approach can be accurately and precisely applied for the estimation of both model drugs for the first-time using nanoparticles in their raw materials and pharmaceutical tablets and can be applicable in quality control laboratories.

## Experimental

### Apparatus and characterization

The spectroscopic measurements were recorded using a Shimadzu UV–Visible spectrophotometer model 1601 (Kyoto, Japan). The spectrofluorimetric measurements were performed by means of Cary Eclipse Fluorescence Spectrophotometer (Agilent Technologies, USA). The apparatus was equipped with a Xenon flash lamp and the voltage was set at 800 V. A smoothing factor of 19 and a slit width of 5 nm were applied during the study. For controlling and observing the temperature, an England Cambridge Ltd. Shaker water bath was operated. A Consort P-901 pH-meter (Belgium) was utilized for pH measuring and adjustments. An ultrasonic model SS 101 H 230 (USA) was also utilized for sonication. The morphology and size of the green fabricated particles was investigated by Transmission electron microscope (TEM) images. The examination was done at 200 kV using JSM-2100 Transmission electron microscope (JEOL, Tokyo, Japan) with Samples loaded at carbon coated 200 mesh Cu-grid. The measurement of zeta potential of Ag-NPs was conducted using a Malvern Panalytical Zetasizer (Workcestershire, UK). FTIR analysis is conducted using JASCO Ft/ir-4100 Fourier Transform Infrared Spectrometer. EDX analysis was performed using energy-dispersive X-ray spectroscopy attached to scanning electron microscope (JEOL, JSM-IT100 LA).

### Chemicals and materials

RIV with purity of 99.94% was kindly obtained from Memphis Co. for pharm., Cairo, Egypt. EDT with purity 99.8% was kindly supplied by NODCAR, Cairo Egypt. Rivarospire® tablet, batch no. 222120, claimed to contain 2.5 mg per tablet, product of Inspire pharmaceutical company, 4 El Tahrir st, Sheraton Bldgs, Cairo Egypt and Coaguloban® tablets batch no.2230658, claimed to contain 30 mg per tablet, Maryrl Pharmaceutical industries El Obour City, The West Extension, Block 20005, Cairo, Egypt were purchased from community pharmacy in Mansura, Egypt. Silver nitrate with purity of 99.8%, boric acid and methanol (HPLC grade) were bought from Sigma Aldrich (USA). Glacial acetic acid (96%), phosphoric acid and Sodium hydroxide were obtained from El-Nasr Pharmaceutical Chemicals Co., Cairo, Egypt. Chemicals with analytical grade and distilled water were consumed throughout the study.

### Standard stock solutions, reagents and buffer solutions

A quantity of 0.01 g from each drug was accurately weighed, transferred to a 100 ml measuring flask, and dissolved in 20 ml methanol, then subjected for sonication for 5 min. A stock solution (100 μg/ml) for each drug was obtained by completing to the mark with methanol. Working solutions were prepared by dilution with distilled water as diluting solvent as appropriate. An aqueous AgNO_3_ solution, 1 mM was prepared. Britton Robinson buffer solutions were prepared by mixing 0.04 M of each of the three acids: acetic acid, boric acid, and phosphoric acid. The intended pH range (2–12) of buffer solutions was adjusted with 0.2 M solution of sodium hydroxide (NaOH).

### Preparation of orange peel extract

Fresh orange (citrus sinensis) fruits were purchased from a local fruit seller in Mansura, Egypt. Fresh orange peels were isolated from the fruits, washed thoroughly with tap water and then with distilled water to strip off any debris or unwanted substances, then cut into small pieces. Accurately weighed 4 g of finely sliced peels were then transferred into a 250 ml beaker containing 40 ml of distilled water and then subjected for boiling for about 2 min after proper mixing. After that, with the aid of Whatman No.1 filter paper, the yielded extract was filtered, and the isolate was then collected in a 50 ml measuring flask. That filtrate was kept in the refrigerator for further use.

### Biogenic synthesis of silver nanoparticles (AgNPs)

The biogenic fluorescent Ag-NPs were fabricated via adopting an environmentally benign synthesis approach without using harsh or toxic chemicals [59]. Ag-NPs were prepared by adding 3 ml of the extract to 40 ml of 1 mM silver nitrate solution and left at room temperature for approximately 1 h. The color of the solution turned yellowish brown in 20 min and the color became deeper with rise in time. The final brown color was obtained after 1 h when the reduction was completed, and further time did not lead to any change in color. The prepared nanoparticles' solution was kept in amber-colored bottle in the refrigerator at 4⁰C for further use and remained stable for at least 2 months with no noticeable alteration in its analytical physical or chemical properties.

### Measurement of fluorescence quantum yield of biogenic Ag-NPs

The fluorescence quantum yield of the biogenic Ag-NPs was measured using solution as a reference standard. The reference solution of 2-amino pyridine was prepared 0.1 M H_2_SO_4_ aqueous solution with quantum yield (${\Phi }_{{\varvec{S}}{\varvec{t}}}$) corresponds to 0.6. The subsequent equation was applied for the estimation of quantum yield Φ of Ag-NPs [60]:

1 $${\varvec{\varPhi}}_{{{\varvec{NPs}}}} ={\varvec{\varPhi}}_{{\varvec{S}}} \times \left( {\frac{{{\varvec{F}}_{{{\varvec{NPs}}}} }}{{{\varvec{F}}_{{{\varvec{St}}}} }}} \right) \times \left( {\frac{{{\varvec{A}}_{{{\varvec{St}}}} }}{{{\varvec{A}}_{{{\varvec{NPs}}}} }}} \right) \times \left( {\frac{{{\varvec{\eta}}_{{{\varvec{NPs}}}} }}{{{\varvec{\eta}}_{{{\varvec{St}}}} }}} \right)^{2}$$

where:

${\Phi }_{\text{NPs}}$, ${\Phi }_{\text{St}}$ denotes the prepared Ag-NPs and the standard 2-amino pyridine quantum yield, respectively.

${\text{F}}_{\text{NPs}}$, ${\text{F}}_{\text{St}}$ denotes the integrated FI of Ag-NPs and the reference solutions, respectively after excitation at 258 nm.

${\text{A}}_{\text{NPs}}$, ${\text{A}}_{\text{St}}$ is the absorbance values of Ag-NPs and the reference solutions at 258 nm.η denotes the refractive index of the solvent.

The absorbance values of the standard solutions of 2-amino pyridine and Ag-NPs were maintained less than 0.1 at ${\uplambda }_{\text{ex}}$ to reduce the inner filter effect.

### General procedure for the assay

#### Construction for calibration curves

Into a series of 10 ml volumetric flasks, different aliquots of working standard solutions of RIV and EDT were transferred to obtain the linear concentration range which lies between (0.5–10) µg/ml for both drugs as shown in Table 1. Sequentially, 0.4 ml of Ag-NPs solution was then added, and then completed to the mark with distilled water to attain the studied final concentrations. Fluorescence spectra were measured at 333 nm after excitation at 258 nm. A blank sample processed in the same manner was simultaneously measured. The decrease in fluorescence intensity of Ag-NPs (ΔF) was plotted against final drug concentration (µg/ml) to attain standard calibration curves. The regression equations were then produced. The findings illustrated in Table 2 showed acceptable percentage recoveries.

**Table 1:** Performance data for the analysis of RIV and EDT by the proposed method

Parameter	At 333 nm		At 485 nm	
	RIV	EDT	RIV	EDT
Concentration range (µg/ ml)	0.5–10	0.5–10	1–9	0.7–7
LOD (µg/ml)	0.14	0.16	0.21	0.07
LOQ (µg/ml)	0.44	0.48	0.64	0.21
Correlation coefficient (r)	0.9999	0.9999	0.9998	0.9999
Slope	34.4763	41.2528	57.35	19.94
Intercept	23.91	24.62	112.30	129.20
Sy/x	2.26	1.75	4.06	0.60
S_a_	1.50	1.17	3.69	0.42
S_b_	0.25	0.19	0.64	0.11
% Error	0.39	0.25	0.43	0.12
%RSD	1.04	0.67	0.95	0.29
No. of Experiments	7	7	5	6
Mean found (%) ± SD	99.84 ± 1.04	100 ± 0.67	99.91 ± .95	99.99 ± 0.29

S_y/x_ = standard deviation of the residuals. S_a_ = standard deviation of the intercept of regression line. S_b_ = standard deviation of the slope of regression line. % Error = RSD%/ $\surd n$

**Table 2:** Application of the proposed method to the determination of RIV and EDT in their raw materials

Compound	Proposed method						Comparison method		
	At 333 nm			At 485 nm					
	Amount taken (μg/ml)	Amount found (μg/ml)	%Found*	Amount taken (μg/ml)	Amount found (μg/ml)	%Found*	Amount taken (μg/ml)	Amount found (μg/ml)	%Found*
Rivaroxaban	0.5	0.49	98.00	1	0.99	99.00	5	5.01	100.20
	1	0.99	99.00	3	2.97	99.00	7	6.91	98.71
	3	3.04	101.33	5	5.08	101.60	10	10.10	101.00
	5	4.97	99.40	7	7.00	100.00	15	14.96	99.73
	7	7.06	100.86	9	8.96	99.56			
	9	8.91	99.00						
	10	10.05	100.50						
X^−^ ± SD			99.72 ± 1.20			99.83 ± 1.07			99.91 ± 0.96
Student's t test			0.29 (2.26) **			0.11(2.36) **			
Variance ratio (F test)			1.56(8.94) **			1.23(9.12) **			
Edoxaban	0.5	0.50	100	0.7	0.69	98.57	4	4.03	100.75
				1	0.99	99			
	1	1.000	100.00	2	2.00	100.00	6	5.99	99.83
	3	2.99	99.67	3	3.01	100.33	10	10.06	100.60
	5	4.98	99.60	5	5.00	100.00			
	7	7.07	101.00	7	6.98	99.71			
	9	8.90	98.88						
	10	10.05	100.50						
X^−^ ± SD			99.95 ± 0.68			99.60 ± 0.68			100.39 ± 0.49
Student's t test			1.11(2.26) **			1.23(2.45) **			
Variance ratio (F test)			1.91(19.33) **			1.91(19.25) **			

^*^Each result is an average value of three separate determinations

^**^Values between parentheses are the tabulated t and F values respectively, at *p* = 0.05

#### Procedure for estimation of pharmaceutical dosage forms

Ten Rivarospire® tablets and ten Coaguloban® tablets were accurately weighed, gently crushed and mixed. An accurate quantity of each powder equivalent to 0.01 g of RIV and EDT was then transferred into 100 ml measuring flasks and approximately 40 ml methanol was added to each flask. The mixture was then sonicated for 30 min and completed to the final mark with the same solvent. The obtained solutions were filtered by Whatman No.1 filter paper into 100 ml measuring flasks whilst discarding the first milliliters of the filtrate. Different volumes of the filtrate were transferred into a set of 10 ml volumetric flasks followed by addition of 0.4 ml of Ag-NPs solution and the volumes were completed to the mark with distilled water. The obtained working solutions covering the concentration range for each drug were assayed by following the prescribed general procedure under 2.7.1 section. The nominal contents of tablets and standard deviations were calculated by applying the corresponding regression equations. Results are presented in Table 3.

**Table 3:** Application of the proposed method for determination of RIV and EDT in their pharmaceutical dosage forms

Compound	Proposed method			Comparison method		
	Amount taken (μg/ml)	Amount found (μg/ml)	%Found*	Amount taken (μg/ml)	Amount found (μg/ml)	%Found*
Rivarospire 2.5 mg	3	3.04	101.33	3	3.02	100.66
	5	4.98	99.60	5	4.96	99.20
	7	7.05	100.71	7	6.89	98.43
X^−^ ± SD			100.55 ± 0.88			99.43 ± 1.13
Student’s t test			1.35 (2.78)**			
Variance ratio (F test)			1.67 (19.00)**			
Coaguloban 30 mg	1	1	100	4	3.96	99.00
	3	3.02	100.66	6	6.02	100.33
	7	7	100	8	8.05	100.63
X^−^ ± SD			100.22 ± 0.38			99.99 ± 0.87
Student’s t test			0.43 (2.78)**			
Variance ratio (F test)			5.18 (19.00)**			

*Each result is an average value of three separate determinations

**Values between parentheses are the tabulated t and F values respectively, at *p* = 0.05

## Results and discussion

### Green biogenic synthesis of plant-rooted Ag-NPs

In this paper, Ag-NPs were impressively prepared adopting a green, a bio-inspired, biocompatible, facile, environmentally begin and cost-effective procedure instead of relying on costly and hazardous chemicals. Moreover, bioinspired synthesis provides many advantages executing its dominance over other chemical and physical synthetic methods. It eliminates the use of toxic and expensive chemicals that are detrimental to both humans and the environment. It also eliminates high-energy consumption in comparison with physical methods. Our approach relied on the synthesis from renewable, naturally occurring, sustainable, economical, and available starting materials. These advantages are consistent with the green chemistry principles and achieve the concept of circular economy and sustainability.

**Fig. 2:**
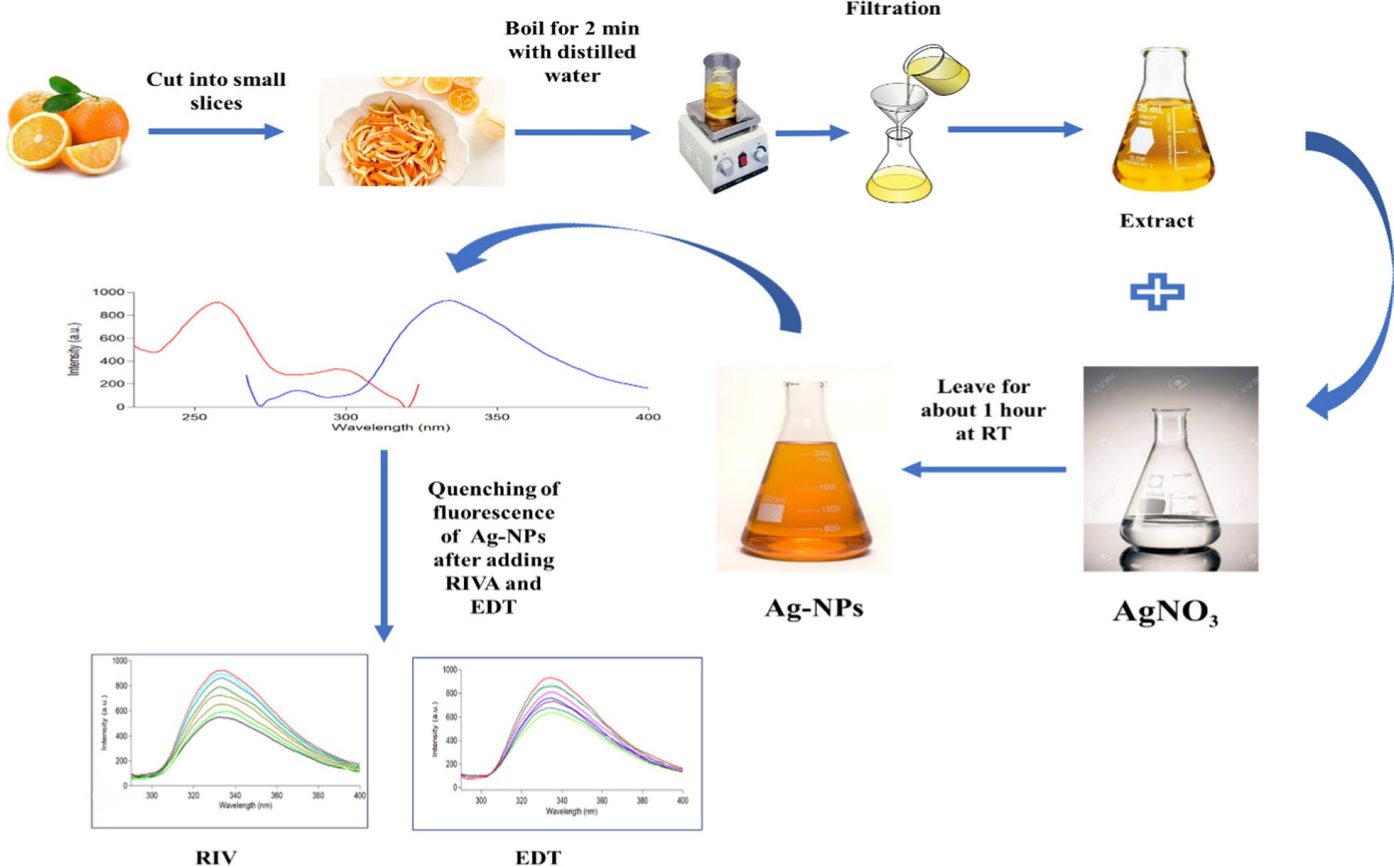
Schematic outline for the synthesis of the fluorescent bioinspired Ag-NPs

Additionally, plant-based synthesis of Ag-NPs provides integrated synthesis process as different biomolecules such as flavonoids, ketones, phenolic and carboxylic acids, aldehydes, and tannins play an interconnected role during the nanoparticle synthesis. They do not act only as reducing agent for reduction of Ag^+^ to Ag^0^, but also, they act as stabilizing agent eliminating the need of expensive chemical stabilizing agents [61]. In our study, we utilized the waste of orange (orange peel) as renewable, sustainable, and eco-friendly bio-occurring material instead of resembling a load polluting the environment, that in turn encourages recycling of materials and reduce the dependence on non-renewable and depleting resources. The abundance, availability, and low price of orange peel in all areas around the world enable the comprehensive practicability of the suggested approach. Figure 2 represents the schematic outline for the steps for biogenic synthesis of Ag-NPs in the designed study.

### Morphological and structural characterization of the biogenic Ag-NPs

**Fig. 3:**
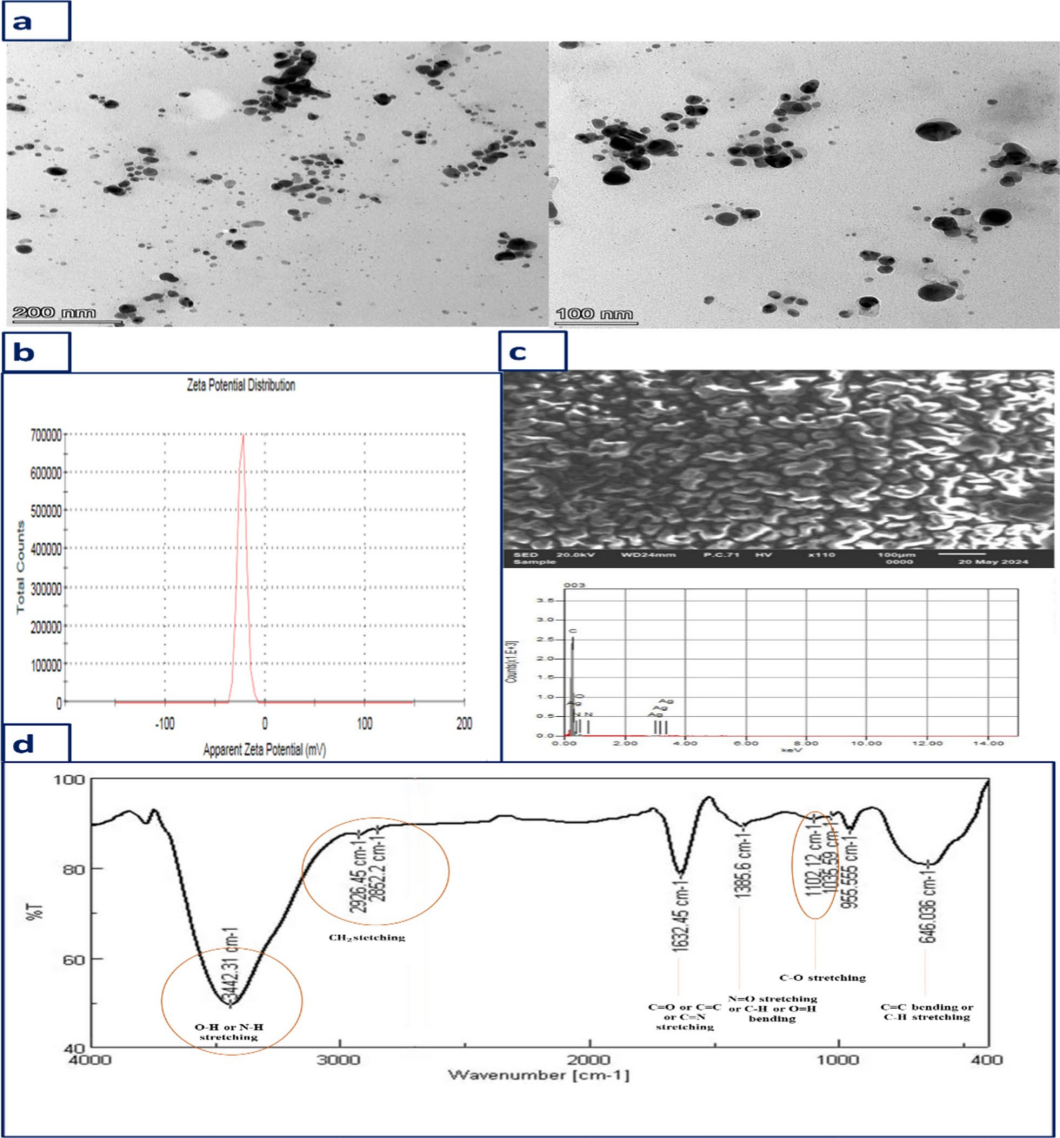
**a** TEM image of Ag-NPs ($9.3\times {10}^{-4})$ M, **b** Zeta potential distribution curve, **c** EDX spectrum and **d** FTIR of the prepared Ag-NPs

The comprehensive characterization of Ag-NPs had been accomplished through different microscopic and spectroscopic techniques. By using TEM image, the size and morphological properties of fluorescent Ag-NPs were examined. Figure 3a represents TEM image of Ag-NPs, which are mono dispersed sphere-shaped nanoparticles. Their size ranges from 11.6 to 15.7 nm. Furthermore, for better elucidation of the surface charge and stability of the prepared Ag-NPs, zeta potential was measured. Zeta potential (ζ) is a crucial hallmark of nanoparticles. It is considered as a measure of the electrostatic repulsion of negatively charged particles in the dispersed system. It was confirmed to equal −23 mV as shown in Fig. 3b. As known the higher zeta potential (ζ), the higher the stability of the colloid system, so the prepared nanoparticles imply good stability. The EDX spectrum shown in Fig. 3c confirmed the formation of Ag-NPs by displaying a silver absorption signal at 3 kV. FTIR is deemed as an essential means for the exploration of chemical composition of the surface of Ag-NPs. Figure 3d demonstrates the surface functional groups of Ag-NPs. The strong absorption peak at 3342 cm^−1^ is allocated for alcohol (O–H) or amide (N–H) stretching. The peaks at 2926 and 2852 cm^−1^ are attributed to stretching of the methylene (${\text{CH}}_{2}$). While the narrow absorbance peak at 1632 cm^−1^ is associated with carbonyl (C=O) of aldehydes or ketones, alkene (C=C) or imine (C=N) group stretching. The absorption peak at 1385 cm^−1^ is attributed to nitroso (N=O) group stretching, (C–H) bending or (O–H) bending. The peaks obtained at 1102, 1035 cm^−1^ are assigned to C–O stretching. The absorption bands at 955 and 646 cm^−1^ are related with (C=C) bending or (C–H) stretching of alkene. The prevalence of aldehyde or ketone groups adsorbed at the surface of nanoparticles confirms the reducing ability of the extract and their capability to bind with Ag-NPs as a capping or stabilizing agents in addition to enhancing their water solubility and polarity.

### Optical and spectral characteristics of Ag-NPs

**Fig. 4:**
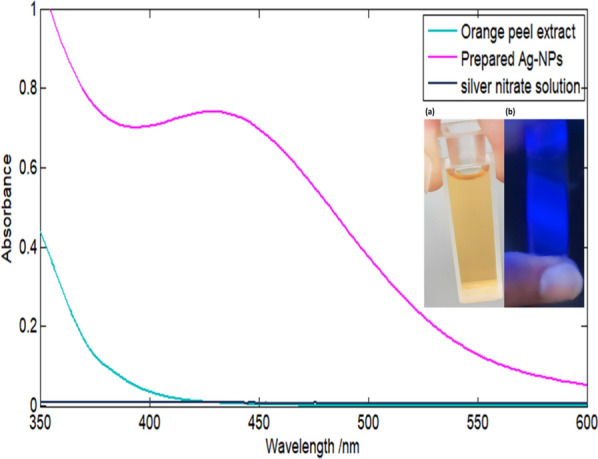
UV–Visible spectrum of **a** the bio-synthesized Ag-NPs $( 9.30\times {10}^{-4})$ M and its starting materials with inset optical images of Ag-NPs under **a** normal day light and **b** UV light

The UV–Vis absorption spectrum of Ag-NPs reveals characteristic absorption band at about 430 nm verifying the development of Ag-NPs. As noticed, Fig. 4 explains the UV–Vis spectrum of Ag-NPs and its initial synthesis materials, silver nitrate solution and orange peel extract. The prominent absorbance in UV–Vis region is accompanying the localized surface plasmon resonance phenomenon of Ag-NPs that is absent in the spectrum of bulk metal. The bio fabricated Ag-NPs solution showed blue fluorescence under UV lamp as shown in Fig. 4. Optical features of Ag-NPs, which are size reliant, emerge from interband and intraband electron transitions, the induced magnetic field of oscillations of collective band electrons and surface dispersion [62, 63].

**Fig. 5:**
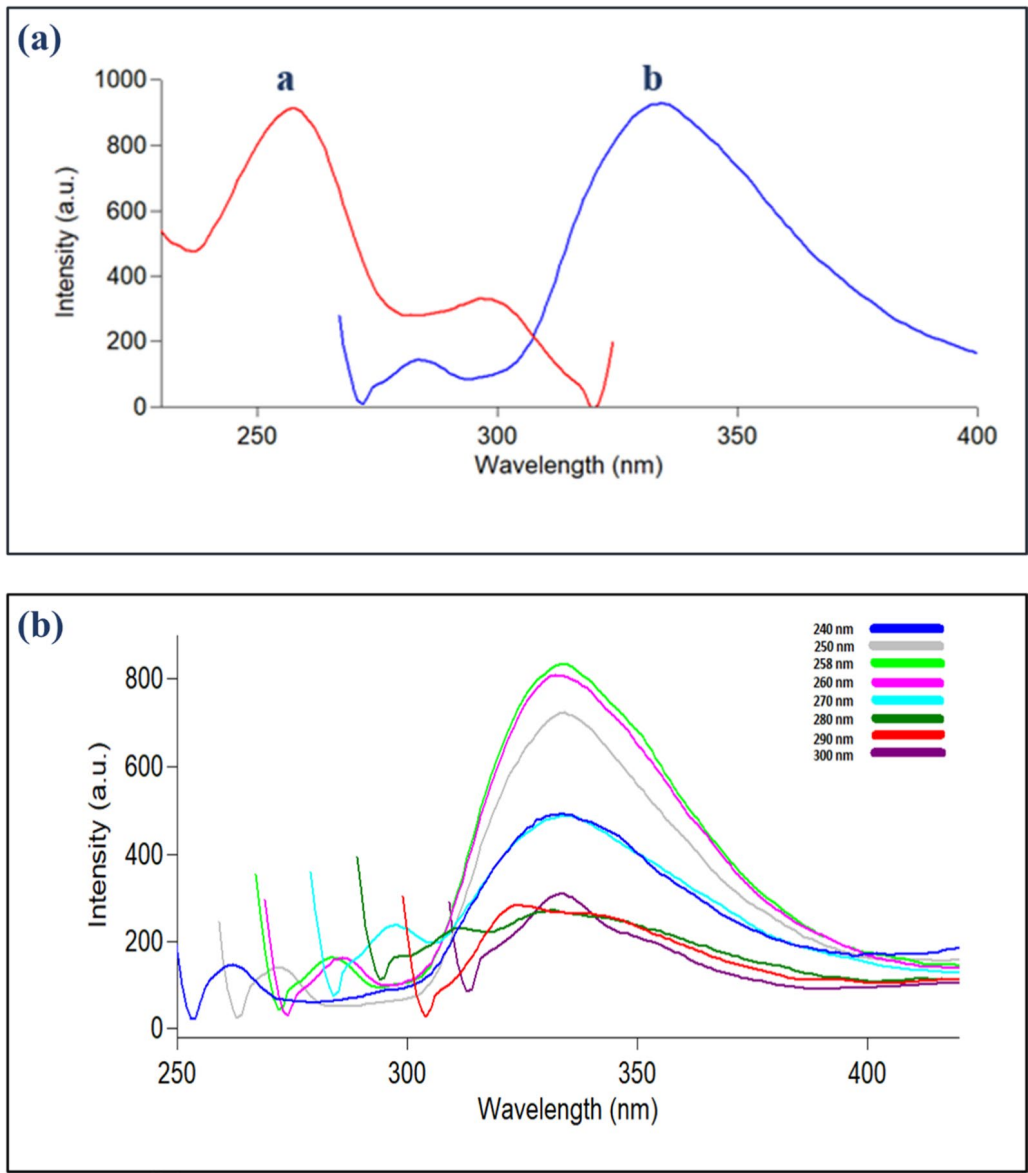
**a** Fluorescence spectra of the biosynthesized Ag-NPs $( 3.72\times {10}^{-5})$ M where *a* and *b* represent excitation and emission spectra at 258 and 333 nm, respectively, **b** emission behavior of Ag-NPs upon varying λ_ex_ from 240–300 nm

The fluorescence bands of Ag-NPs are revealed in Fig. 5a, and they exhibited strong emission at 333 nm after being excited at 258 nm. Upon differing the excitation wavelength from 240 to 300 nm, it was observed that the emission spectra are consistent $({\uplambda }_{\text{em}}=333\text{ nm})$ and is not wavelength dependent (Fig. 5b). This confirms consistent energy relaxation behavior to a single ground state of the prepared nanoparticles. The emission at 333 nm showed higher sensitivity and wider linear range than the other emissive peak at 485 nm after excitation at 242 nm, so it is selected during the study as depicted in Tables 1 and 2 [64]. Although, the chemical synthesis procedure took less time than the green synthetic one. The core difference between the chemically and biogenic synthesized Ag-NPs is the facile, environmentally begin and cost-effective synthesis procedure instead of relying on costly and hazardous chemicals (sodium borohydride). The prepared Ag-NPs revenue a quantum yield $(\Phi$) of 17.15% using 2-amino pyridine as a reference standard inferring promising fluorescence merits.

On the basis of the importance of the stability of fluorescent substances, the stability of Ag-NPs was checked carefully. The solution of Ag-NPs manifests great stability for nearly 5 months without any conspicuous alteration in form of precipitation, color change, turbidity and floating which confirmed by zeta potential measurement. It retained 96% of its initial fluorescence capability.

**Fig. 6:**
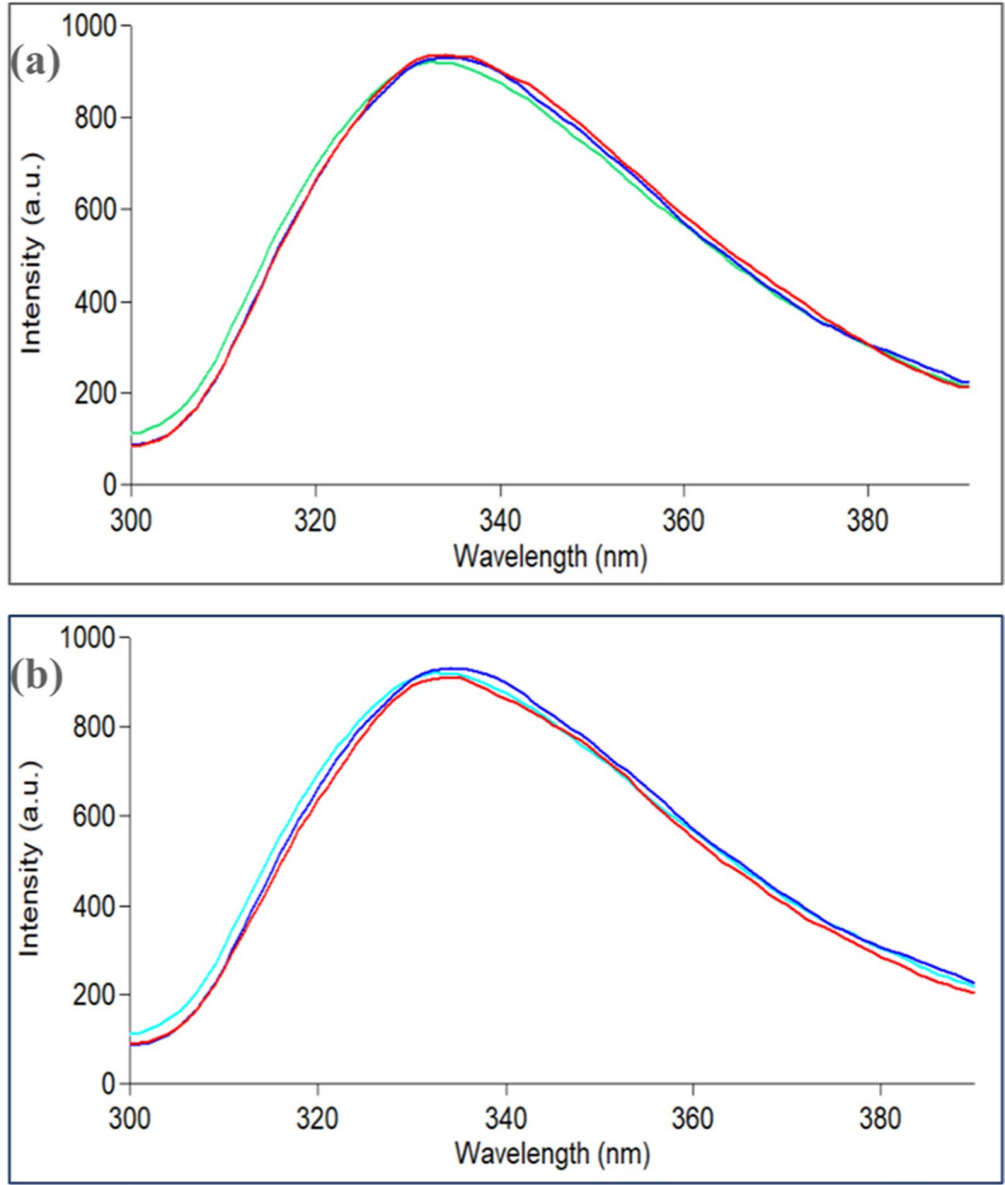
Fluorescence emission spectra of Ag-NPs ($9.30\times {10}^{-4})$ M using **a** orange collected in different spaced periods, **b** orange collected from different regions

The replicability and validity of the developed study is established by using different varieties of orange. Orange is bought over different spaced periods (March, April, and May) and from different regions in Egypt and utilized for the synthesis procedure. It is observed that no change in the fluorescence wavelengths has been detected as illustrated in Fig. 6. Furthermore, the relatively low values of relative standard deviations (%RSD) among individually synthesized preparations of Ag-NPs from orange collected in different spaced periods and orange collected from different regions (1.87, 1.78%, respectively) ensured the resilience of the synthetic approach and its repeatability in many different regional laboratories.

Concisely, our presented fluorescent probe Ag-NPs combines significant traits such as high-water solubility, high quantum yield, optical properties and excellent stability granting its feasible application in drug sensing. Besides, the integrated green method, whether the synthesis procedure or the reducing agent, surpasses other traditional synthetic methods. It was unequivocal that the fluorescent sensor is fabricated from renewable, sustainable, and eco-friendly available material that in turn encourages recycling of useless and throwable materials, minimizes extensive consumption of depleted materials, eliminates the usage of hazardous and expensive solvents and chemicals, and reduces the energy expenditure. Those comprehensive qualities confer green and superior applicability of the suggested sensing probe.

### Explanation of fluorescence quenching process

**Fig.7:**
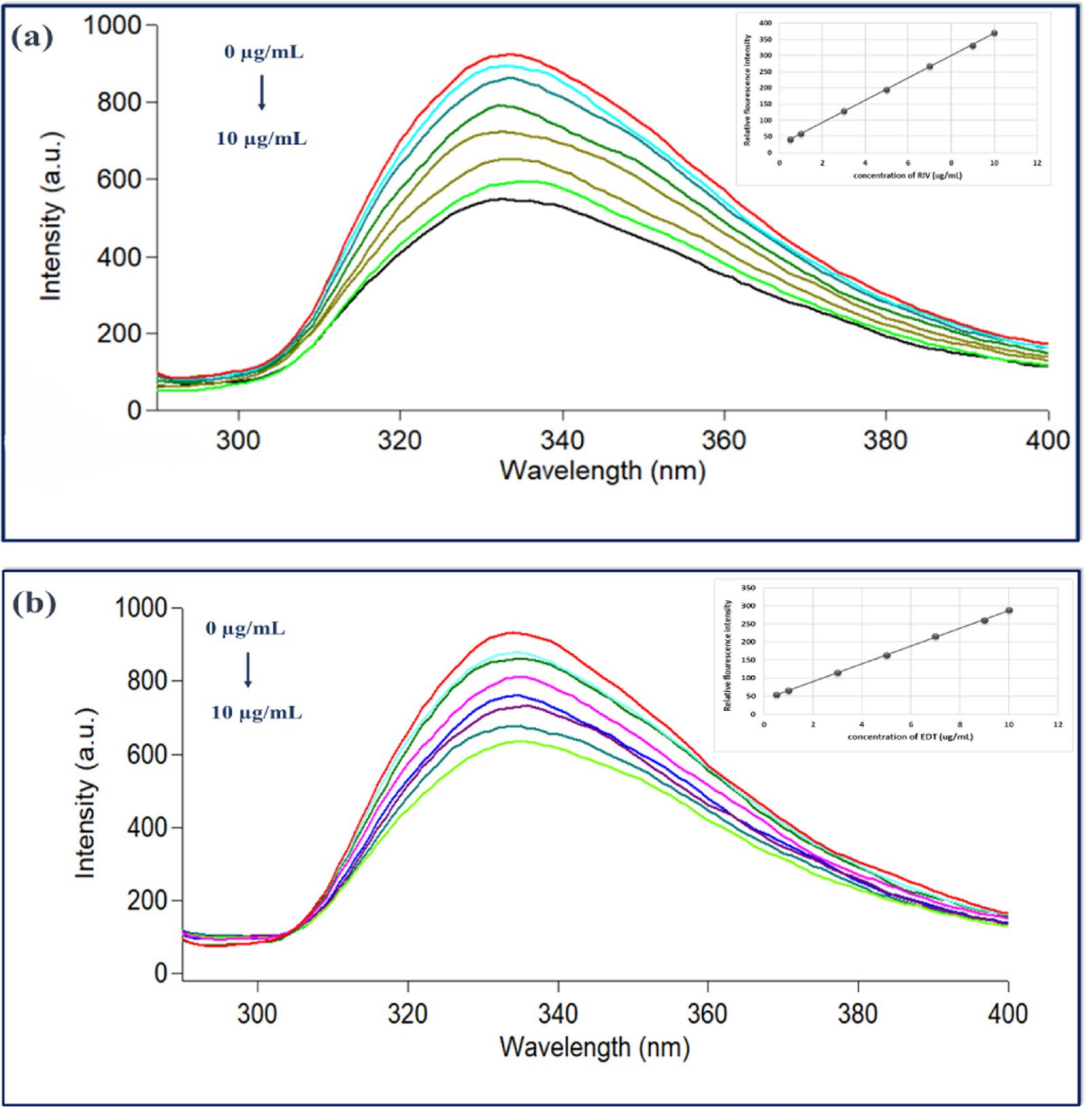
Fluorescence spectra of Ag-NPs $\left(3.72\times {10}^{-5}\right)$ M in aqueous solution upon addition of various concentrations of **a** RIV (from *top* to *bottom*: 0, 0.5, 1, 3, 5, 7, 9 and 10 µg/ml) and the *inset* shows the corresponding calibration curve between RFI and drug concentration **b** EDT from *top* to *bottom*: 0, 0.5, 1, 3, 5, 7, 9 and 10 µg/ml) and the *inset* shows the corresponding calibration curve between RFI and drug concentration

**Fig. 8:**
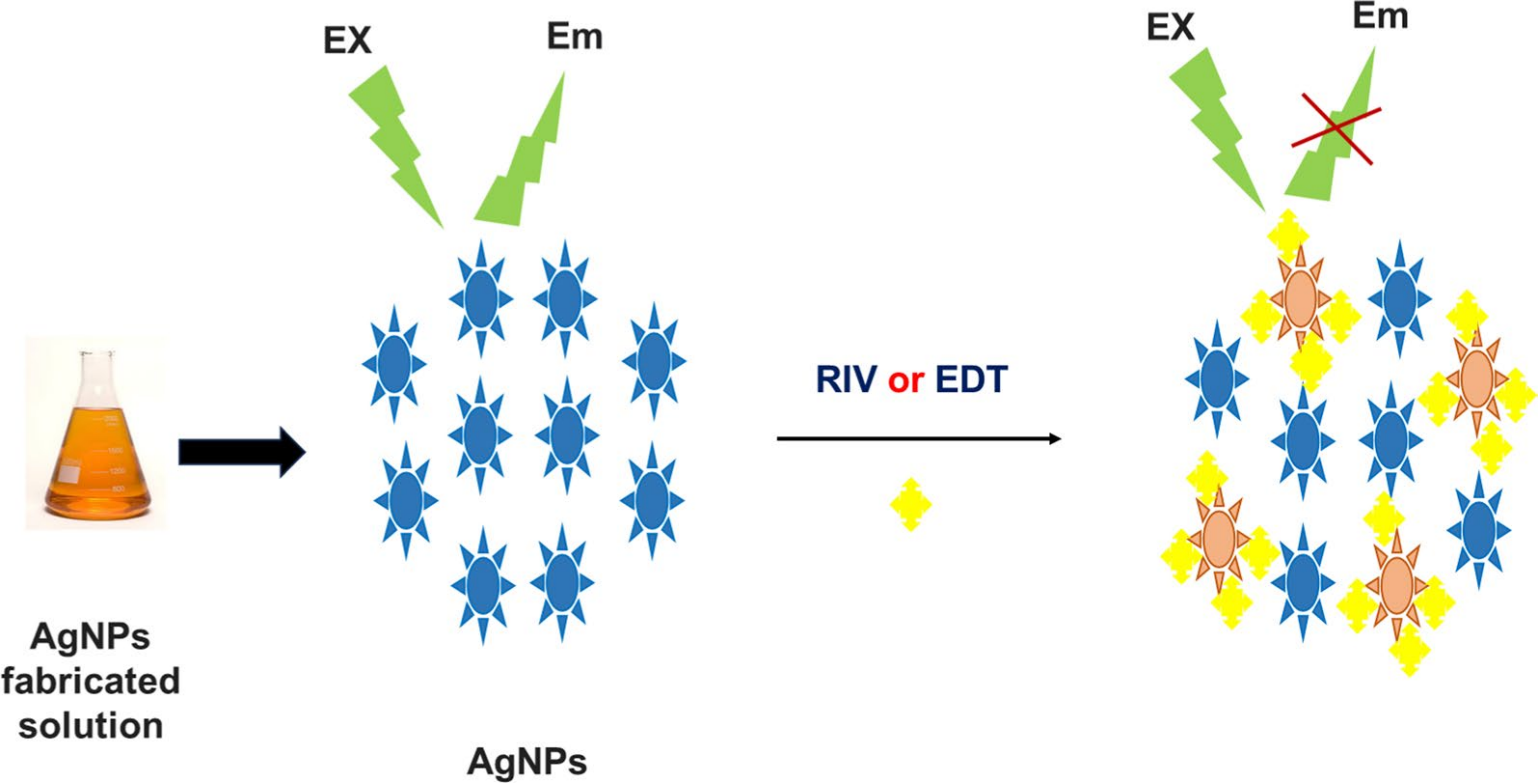
schematic diagram of the quenching process of Ag-NPs by the studied drugs

**Fig. 9:**
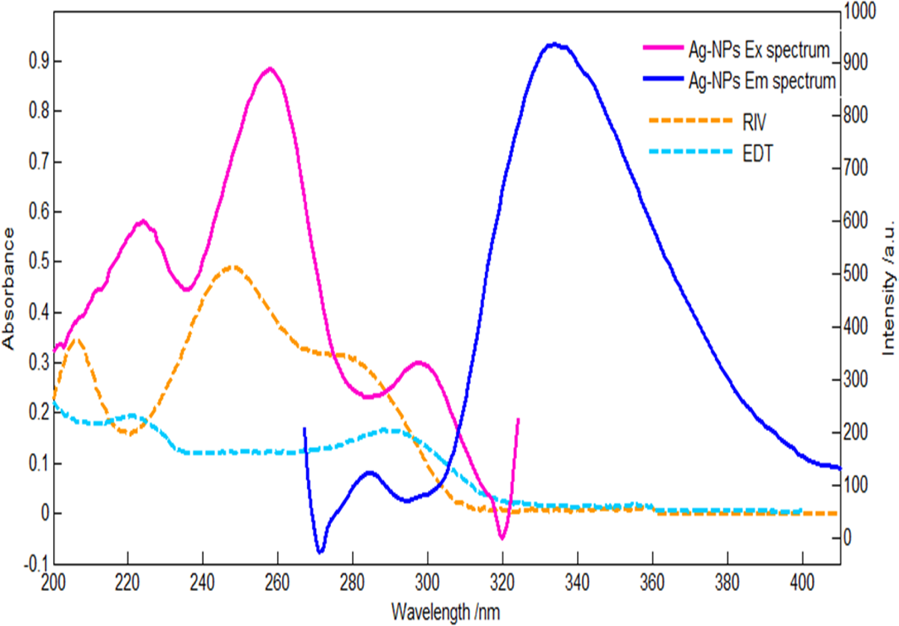
A co-plot of slight spectral overlap between UV-absorption spectra of RIV (10 µg/ml) and EDT (5 µg/ml) and the excitation and emission fluorescence spectrum of Ag-NPs

The emission peak of Ag-NPs at 333 nm is progressively reduced upon addition of various concentrations of the investigated drug under optimal experimental conditions. Figure 7 represents the quenching behavior of fluorescence intensity of Ag-NPs prior and after adding increasing concentrations of each drug. Figure 8 represents schematic diagram for quenching of Ag-NPs by the studied drugs. The fluorescence peak was precisely measured at 333 nm after excitation at 258 nm. Fluorescence quenching phenomenon has been established as a largely studied phenomenon regarding the interaction between biochemical materials. The quenching of fluorescence proceeds on account of any means that lower the fluorescence of any substance [65]. These means can be compiled in dynamic quenching, static quenching, Forster resonance energy transfer (FRET), energy transfer and inner filter effect [66]. It is obvious as demonstrated in Fig. 9 that the UV absorption spectrum of each of RIV and EDT revealed significant overlapping with the excitation spectrum of Ag-NPs, thus energy transfer in the form of primary and secondary inner filter effect (IFE) may contribute to the quenching process. Primary and secondary IFE originate from absorbance of the excitation energy by various chromophores in the solution and the absorbance of fluorophores to their emission radiation correspondingly [66]. Additionally, IFE-based detecting approaches require the presence of an overlap between the absorption spectrum of the quencher with either or both excitation or the emission spectrum of the florescent material. For exploring the quenching mechanism, correction of the observed fluorescence signals of Ag-NPs for probable IFE was examined upon adding the studied drugs by adopting the following Eq. (2).

2 $${\mathbf{F}}_{{{\varvec{corr}}}} = {\varvec{F}}_{{{\varvec{obs}}}} \times 10^{{\left( {{\varvec{Aex}} + {\varvec{Aem}}} \right)/2}}$$

where:

${\text{F}}_{\text{corr}}$ implies the corrected fluorescence intensity of Ag-NPs after eliminating IFE from observed fluorescence.

${\text{F}}_{\text{obs}}$ is observed fluorescence intensity the Ag-NPs.

${\text{A}}_{\text{ex}}$ and ${\text{A}}_{\text{em}}$ refer to the prospective absorbance values of the quencher (either of RIV or EDT) at ${\uplambda }_{\text{ex}}=258\text{ nm}$ and ${\uplambda }_{\text{em}}= 333\text{ nm}$ of Ag-NPs.

Afterwards, the efficiency of the suppressed fluorescence (%E) for both the observed and corrected FI was estimated in pursuance of the following Eq. (3).

3 $$\% {\varvec{E}} = \left[ {1 - \left( {\frac{{\varvec{F}}}{{{\varvec{F}}^\circ }}} \right)} \right] \times 100$$

**Fig. 10:**
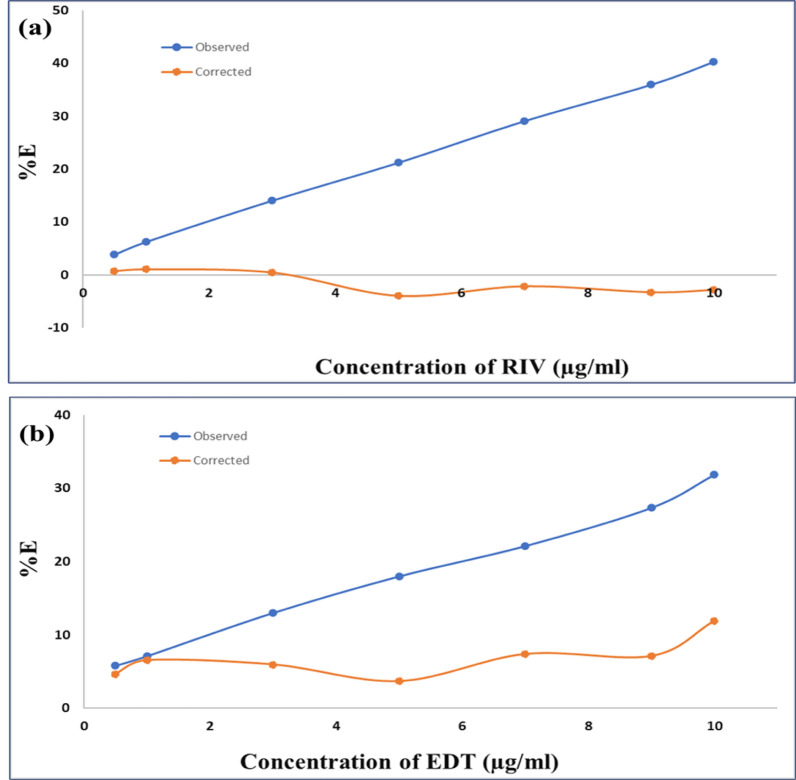
Suppressed efficiency (%E) of observed and corrected fluorescence of Ag-NPs after adding different concentrations of **a** RIV (0.5, 1, 3, 5, 7, 9 and 10 µg/ml) **b** EDT (0.5, 1, 3, 5, 7, 9 and 10 µg/ml)

Figure 10 demonstrates the calculated %E of both observed and corrected FI against different increasing concentrations of RIV and EDT in µg/ml. Consequently, it was obvious that the IFE is involved in the quenching process of Ag-NPs fluorescence via different contributions. For RIV, it is reached that IFE mechanism is the leading reason for the decay of the fluorescence of Ag-NPs as shown in Fig. 10a. While for EDT (Fig. 10b), IFE does not completely participate in the fluorescence quenching but made a significant contribution by about 62% implying the presence of additional quenching mechanism. Accordingly, stern Volmer investigation was performed to reveal the dependency relationship between the quenching and temperature. Three separate temperatures manner was applied for Stern Volmer experiment as stated by the succeeding Eq. (4) [67].

4 $${\mathbf{F}}^\circ /{\mathbf{F}} = 1 + {\mathbf{K}}_{{{\mathbf{sv}}}} \left[ {\mathbf{Q}} \right] = 1 + {\mathbf{K}}_{{\mathbf{q}}} {{\varvec{\uptau}}}^\circ \left[ {\mathbf{Q}} \right]$$

Regarding:

F and F⁰ indicate the recorded fluorescence spectra of Ag-NPs prior and after existence of each drug, correspondingly; ${\text{K}}_{\text{sv}}$ implies quenching constant of Stern–Volmer; [Q] refers to concentration of the quenchers in mole/L; ${\text{K}}_{\text{q}}$ denotes the bimolecular quenching rate constant; τ° is the lifespan of radiation of Ag-NPs.

**Fig. 11:**
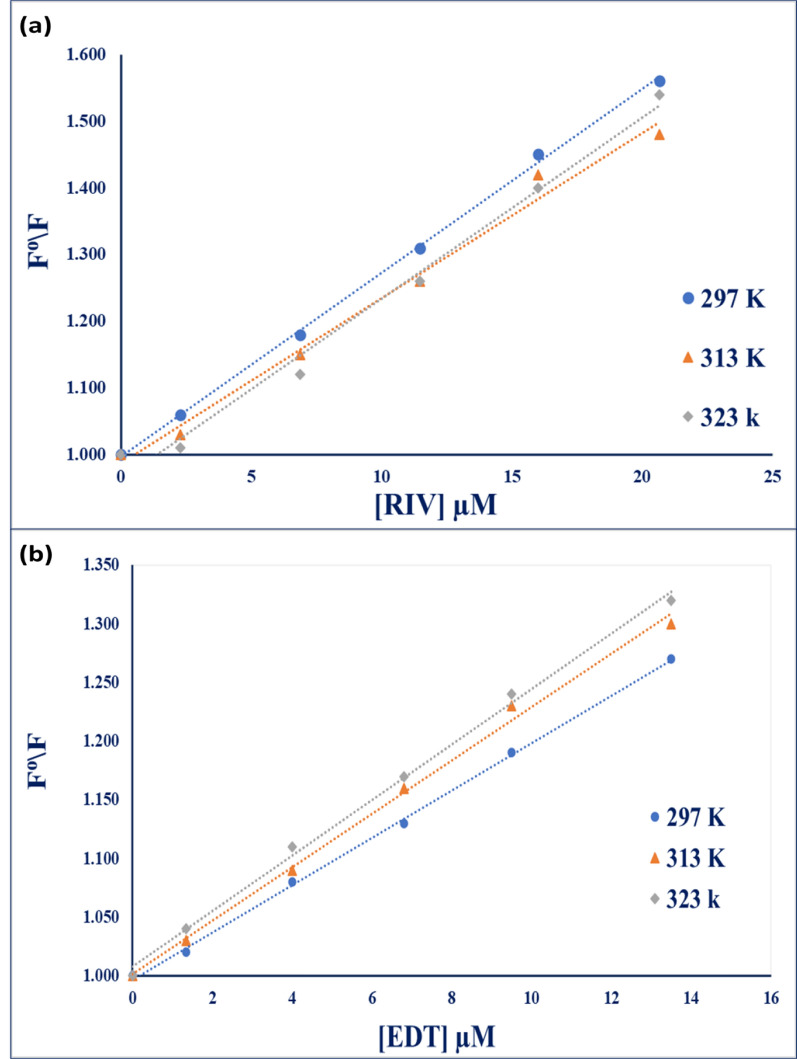
Stern Volmer plot for fluorescence quenching of Ag-NPs for RIV and EDT at different temperatures 297, 313 and 323 K

Stern Volmer experiments were conducted at three rising temperatures 297, 313, 323 K. When plotting F⁰/F versus molar concentration of each drug, a straight line of an intercept equals 1 was obtained as shown in Fig. 11a, b. Upon resolving the Stern–Volmer parameters as illustrated in Table 4, for RIV, ${\text{K}}_{\text{sv}}$ values showed no dependency on the temperature that excluded the participation of static and dynamic quenching in the quenching phenomenon. On the other hand, ${\text{K}}_{\text{sv}}$ values of EDT were increasing upon increasing temperature which is a typical dynamic quenching. Dynamic quenching proceeds from the interaction of a fluorophore in the excited state with the quencher leading the excitation energy being relaxed to the ground state [65].

**Table 4:** A summary of Stern–Volmer parameters of the quenching drugs and Ag-NPs

Drug	Temperature (K)	Stern–Volmer equation (Q, mol)	Correlation coefficient (r^2^)
RIV	Room temperature 297	$F^\circ /F=0.997+2.75\times {10}^{4}[Q]$	0.9990
	313	$F^\circ /F=0.9867+2.48\times {10}^{4}[Q]$	0.9888
	323	$F^\circ /F=0.9622+2.72\times {10}^{4}[Q]$	0.9876
EDT	Room temperature 297	$F^\circ /F=0.9967+2.02\times {10}^{4}[Q]$	0.9990
	313	$F^\circ /F=1.0014+2.28\times {10}^{4}[Q]$	0.9963
	323	$F^\circ /F=1.0079+2.37\times {10}^{4}[Q]$	0.9969

### Optimization of experimental parameters

The effect of different experimental conditions had been studied for better performance of the proposed technique and perceiving the optimal analytical conditions. The explored factors included effect of pH, volume of Ag-NPs and the contact time between the Ag-NPs and the studied drugs.

#### Effect of pH

**Fig. 12:**
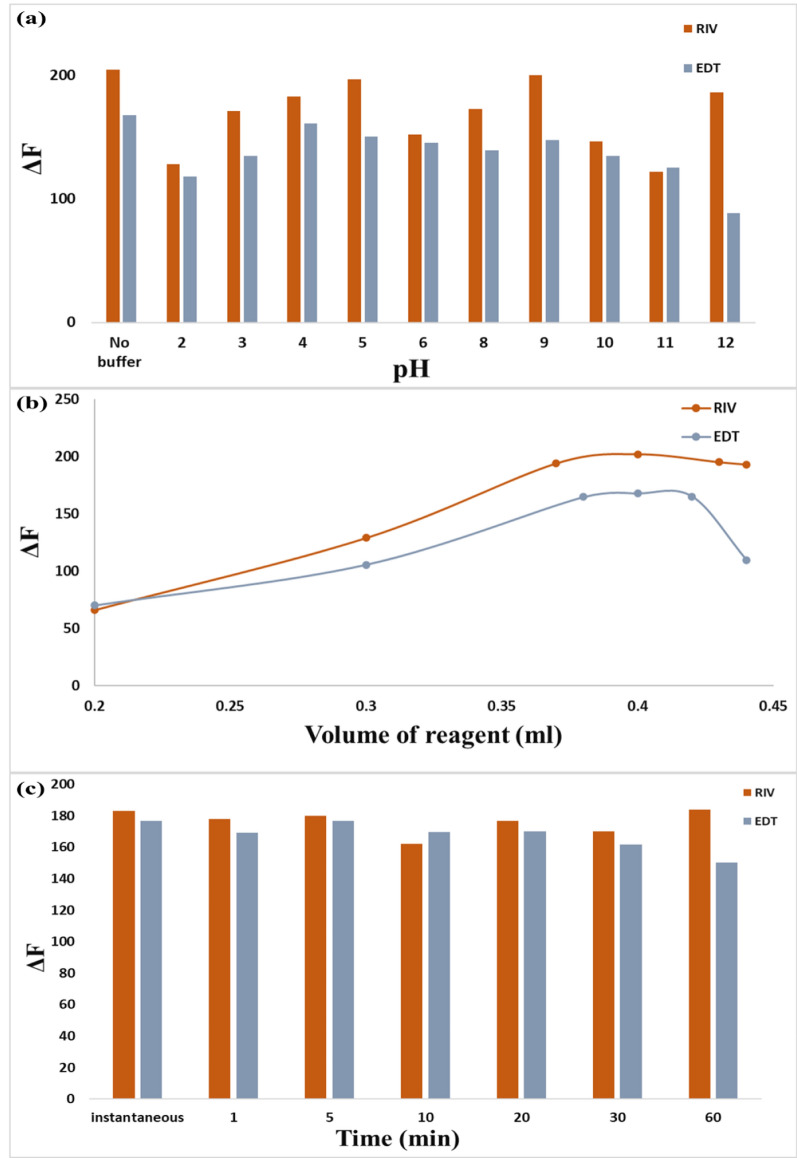
**a** Effect of pH of 1 ml Britton Robinson buffer (0.2 M) pH (2–12) on the fluorescence intensity of Ag-NPs using 5 μg/ml of both RIV and EDT, **b** effect of volume of Ag-NPs (9.3 × 10^–4^ M) on the fluorescence intensity of Ag-NPs using 5 μg/ml of both RIV and EDT, **c** effect of incubation time between Ag-NPs and the studied drugs Ag-NPs using 5 μg/ml of both RIV and EDT

Britton-Robinson buffer (BRB) solutions over the pH range (2–12) were utilized to investigate the impact of pH on the interaction between Ag-NPs and the cited drugs. It was observed that upon changing pH, the quenching performance of fluorescence intensity of Ag-NPs did not show any improvement in comparing with the results using no buffer as shown in Fig. 12a. Consequently, all experiments are conducted without a buffer providing a simpler approach.

#### Effect of volume of Ag-NPs

Different volumes of Ag-NPs solution (9.3×10^−4^) M were studied to obtain the optimum volume that enhanced the performance of the analytical study. It was concluded that 0.4 ml of Ag-NPs gave the maximum degree of quenching (ΔF) for both studied drugs so, it was chosen as the best volume as demonstrated in Fig. 12b.

#### Effect of contact time

To explore the influence of the contact time of the inspected drugs on the quenching efficiency on the fluorescence of Ag-NPs, different incubation periods had been examined. The fluorescence intensities before and after the addition of the studied drugs to Ag-NPs were investigated at time intervals ranging from one min to one hour (1–60 min) as shown in Fig. 12c. It was noted that the reaction between Ag-NPs and the studied drugs was instantaneous and rapid. Moreover, the quenching reaction was immediately complete, and the fluorescence peaks remained steady for around 60 min. Consequently, speed and time savings are crucial features of our study.

#### Validation criteria of the proposed method

The standards of international council for harmonization ICH ${\text{Q}}_{2}{\text{R}}_{1}$ were adhered to guarantee the validity of the recommended approach for routine quality control analysis as clarified in Table 1. The evaluated parameters covered linearity, range, limits of detection and quantification, precision, accuracy, robustness, and selectivity.

#### Range and linearity

The linearity of the suggested technique was investigated by using a serious of various concentrations for both drugs (RIV and EDT) and the fluorescence signals of Ag-NPs were recorded before and after the addition of the two cited drugs. The corresponding standard calibration curves were assembled to reveal the correlation between the degree of reduction of the fluorescence intensity (ΔF) of Ag-NPs and the concentration of each drug. For both RIV and EDT, the relationship was assumed to be linear in the concentration range (0.5–10 µg/ml). Statistical analysis of regression data was performed, and data are depicted in Table 1. The obtained results asserted excellent correlation coefficient (r^2^ = 0.9999) for both drugs and the obtained regression equations were as following:

$$\Delta F = 23.91 + 34.48 C \;\;\;\;\;\;\;\;\;{\text{for RIV}}$$

$$\Delta F = 24.62 + 41.25C {\text{for EDT}}$$

where, C represents the concentration of either RIV or EDT in µg/ml.

Additionally, small, obtained values of standard deviation of slope (${\text{S}}_{\text{b}}$), standard deviation of intercept (${\text{S}}_{\text{a}}$) and residual standard deviation denoted that the method was linear.

#### Detection and quantitation limits

To validate the sensitivity of our suggested technique, LOQ and LOD were determined statistically in compliance with ICH ${\text{Q}}_{2}{\text{R}}_{1}$ regulations as depicted in the following formulae:

$$LOQ = \frac{{10 \times S_{a} }}{b}\;\;\;LOD = \frac{{3.3{ } \times { }S_{a} }}{b}$$

Regarding:

${S}_{\text{a}}$ represents the standard deviation of the intercept while, b represents the slope of calibration graph.

As concluded from LOQ and LOD resulting values shown in Table 1, the anticipated method is adequately sensitive in the estimation of both drugs.

#### Accuracy

Accuracy was examined by comparative assessment the results of our presented study to the results acquired from formerly reported approaches for RIV and EDT respectively in their raw materials and pharmaceuticals [25, 68]. The comparison revealed that was no marked disagreement between the obtained results of the devised and the comparison techniques with respect to accuracy and precision. This was evidenced throughout statistical analysis of the data acquired from the suggested and reported methods employing Student’s t-test and variance ratio F-test as depicted in Table 2 and 3.

#### Precision

Intermediate precision and repeatability were explored to appraise the precision of the technique. Three levels of concentrations from each drug were analyzed in triplicate runs in the same day and over an interval of three consecutive days. These studied drugs showed low values of % RSD (<2%) and % Error (<1%) proves that the method is convincingly precise as abridged in Table 5.

**Table 5:** Inter-day and intra-day precision data for RIV and EDT using the proposed method

Concentration (μg/ml)		Intra-day precision			Inter-day precision		
		Mean ± SD	RSD (%)	Standard error	Mean ± SD	RSD (%)	Standard error
RIV	1	99.88 ± 0.46	0.46	0.27	100.04 ± 0.66	0.66	0.38
	5	99.95 ± 1.00	1.00	0.58	98.96 ± 0.35	0.35	0.20
	7	100.65 ± 0.62	0.62	0.36	100.79 ± 0.17	0.17	0.10
EDT	1	99.64 ± 0.62	0.62	0.36	100.06 ± 0.20	0.20	0.12
	3	99.89 ± 1.44	1.44	0.83	99.71 ± 1.23	1.23	0.71
	7	100.92 ± 0.66	0.65	0.38	99.80 ± 1.46	1.46	0.85

#### Robustness

The estimation of robustness was performed to ascertain the steadiness of the analytical performance of the method by deliberate and minor varies. The slight alteration in volume of Ag-NPs (0.4 ± 0.03 ml), (0.4 ± 0.02 ml) for RIV and EDT, respectively and excitation wavelength (258 ± 2 nm) did not impact the obtained ΔF of both drugs verifying the method's robustness as shown in Table 6.

**Table 6:** Evaluation of the proposed method robustness

	%Recovery ± SD	%RSD
Parameter	RIV	
(a) Volume of reagent Ag-NPs (0.40 ml ± 0.03)		
0.37 ml	99.11 ± 0.56	0.57
0.40 ml	99.95 ± 0.10	0.10
0.43 ml	99.25 ± 1.06	1.05
(b) Excitation wavelength (258 nm ± 2)		
256 nm	101.94 ± 0.92	0.91
258 nm	100.65 ± 0.20	0.20
260 nm	100.63 ± 0.88	0.88
Parameter	EDT	
(a) Volume of reagent Ag-NPs (0.40 ml ± 0.02)		
0.38 ml	100.63 ± 0.38	0.38
0.40 ml	100.39 ± 1.10	1.10
0.42 ml	99.41 ± 1.13	1.14
(b) Excitation wavelength (258 nm ± 2)		
256 nm	99.65 ± 0.93	0.93
258 nm	100.39 ± 1.10	1.10
260 nm	100.32 ± 0.19	0.19

#### Selectivity

The Prescence of additives in the dosage forms may constitute a cause of interference. Consequently, the selectivity was evaluated by analysis of the studied drugs by the recommended method in their pharmaceutical dosage forms. Rivarospire tablet contains the following additives: lactose monohydrate, microcrystalline cellulose, croscarmellose sodium, sodium lauryl sulfate, hydroxypropyl methylcellulose and magnesium stearate. Edoxaban tablet contains the following additives: mannitol, pregelatinized starch, hydroxypropyl cellulose, sodium starch glycolate, microcrystalline cellulose, talc, and magnesium stearate. The high results of percentage recoveries and low values of relative standard deviation and percent error inferred acceptable selectivity of the suggested method. Consequently, excipients did not interfere with the proposed approach.

## Application of the proposed method

### Pharmaceutical application

Our proposed methodology relies on using Ag-NPs as a fluorescent sensor for analysis of RIV and EDT via their fluorescence quenching of a novel biosynthesized Ag-NPs. It was successfully employed for quantitative estimation of those two novel DOACs in their pharmaceutical dosage forms. The formerly derived regression equations were applied for the estimation of % recoveries. The results stated in Table 4 are congruent with those obtained from the previously reported methods [25, 68] for RIV and EDT, respectively. Besides, statistical analysis of the results attained from the proposed technique in comparison with results of the reported methods was accomplished using Student's t-test and variance ratio F-test. It was settled from the calculated t and F values that there are no distinct disagreements between the two approaches with respect to accuracy and precision as represented in Table 4.

### Assessment the greenness profile of the suggested approach

By virtue of chemical accidents and tragedies that the world experienced over the years, green analytical chemistry has garnered much interest. Consequently, there is much necessity for the development of green analytical techniques that pose no risks to both humans and the environment. Moreover, green analytical chemistry does not aim only at minimizing the impact on both humans and the environment, but it also emphasizes on reducing energy consumption, using renewable feedstocks, applying less hazardous chemical synthesis, using safer solvents and increasing energy efficiency. All these criteria have been attained in our developed method.

Many metrics have been designed for evaluating the green character of analytical methods. These metrics help scientists in objective side-by-side comparison between different approaches to determine which method is superior to others depending on their objectives. Old methods such as the National Environmental Methods Index (NEMI) [69] are very general and non-quantitative. It signifies only colors which do not reflect the degree of greenness of the method and cannot be used for comparative assessment of many developed methods. Recently, more quantitative metrics have been developed that overwhelm the downsides of older methods. Additionally, some of them are computerized and easy to use. As a whole, we attempt to assess the greenness of the proposed technique using three different metrics, analytical eco-scale [70], complementary green analytical procedure index (Complex GAPI) [71] and analytical GREEnness calculator (AGREE) [72].

Analytical Eco-Scale, as a semi-quantitative tool, is attempted for greenness assessment of the analytical methodologies. The analytical Eco-Scale is assigned based calculation of total penalty points and then, the acquired penalty points are deducted from 100 which is well-established as ideal scoring of greenness. The whole penalty points are calculated based on parameters and steps regarding the analytical procedures. The estimation of the score relates the quantities of reagents and waste, operational hazards, and the instrumentation energy consumption. Our suggested method score equals 89 implying an excellent greenness [70] as illustrated in Table 7.

**Table 7:** The results for evaluation of the greenness of the proposed method according to the analytical Eco-Scale score, Complex GAPI and AGREE

1. Analytical Eco-Scale final score		
Item		Penalty scores
1. Reagent; amount		
Water		0
Silver nitrate < 1 g		8
2. Instrument		
Spectrofluorometer	<0.1 KW h per sample	0
3. Occupational hazard	Analytical process hermitization	0
4. Waste	No treatment	3
Total penalty points		∑11
Analytical Eco-Scale final score		89
2. Complex GAPI	3. AGREE	
	

Alongside, a more recent and quantitative tool, Complementary green analytical procedure index (Complex GAPI) [71] has been designed for estimating of the greenness profile of the analytical techniques. It is regarded as an expansion of GAPI metric by adding a further hexagonal component at the bottom, as well as the widely known five pictograms. The additional hexagonal component is concerned with the green feature of pre analysis procedures. It enfolds traits such as conditions, chemicals and solvents, equipment, set-up, and final-product purification. The metric focuses on assessment and weighing the impact of each phase on the pre-analysis process, as well as the analytical technique on both the environment and humans. It relies on using a three-color evaluation pattern, and each pictogram is segregated into subdivisions. The green color is corresponding to minimal impact and is filled if particular requirement is encountered. While yellow and red fields represent moderate and high impact associated with different steps prior and during the analytical process. The attained pictogram shown in Table 7 displays the greenness evaluation of both the synthesis of Ag-NPs and the developed approach.

Likewise, Analytical GREEnness calculator (AGREE) [72] is a wide-ranging, user-friendly and computational tool that assess the analytical procedure taking into account the 12 principles of. The overall estimation is governed by the size and sample throughput, energy expenditure, waste formation, amount and toxicity of materials, position of instrumental device and the number of steps of chemical processes. The overall evaluation score is the outcome of the individual result of each principle. The final score ranges from 0–1 in a clock-like graphical representation with color manifestation and whole score and in the middle as shown in Table 7. The score was found to be 0.81 revealing that the method is excellent green and compromises little environmental hazard.

## Conclusion

Our suggested study introduced a new promising optical sensor that was biosynthesized via cost-effective, biocompatible, environmentally benign, and facile procedure. The method relied on fabrication of the Ag-NPs using orange peel extract as bio reducing and capping agents eliminating the need of expensive and toxic reducing and stabilizing agents. Comprehensive characterization was achieved by UV–Vis absorption and fluorescence, IR spectroscopy, energy dispersive X-ray spectroscopy (EDX), TEM image and zeta potential. They possess remarkable supremacy because of their high-water solubility, low toxicity and good optical and physicochemical properties permitting their effective applications in chemical detection of drugs and biomolecules. The plant mediated synthesized Ag-NPs are successfully applied for fluorescence nanosensing of two significant DOACs; RIV and EDT in their raw materials and pharmaceutical tablets. The principle of the study is based on the quantitative quenching of the intense fluorescence of Ag-NPs at 333 nm. The quenching mechanisms were thoroughly investigated shedding light on the intramolecular reflexes of excitable fragments with a nearby accessible energy. It is considered to be the first direct spectrofluorimetric sensing method based on nanoparticles for analysis of both drugs and does not require any pre-derivatization steps. The proposed technique offers straightforward merits such as sensitivity, facile synthesis of the sensor from available, non-expensive, bio-derived and sustainable materials and economic and greenness feature. Additionally, three different metrics agreed upon the greenness outline of the proposed approach. The method offers potential applicability in quality control laboratories and pharmaceutical companies with high accuracy and precision and adequate % recoveries. Besides, the current study encourages the forthcoming use of renewable and bioinspired precursors instead of the reliance on expensive and harmful chemicals in pharmaceutical and biomedical applications.

## Data Availability

The datasets used and/or analyzed during the current study are available from the corresponding author on reasonable request.
